# The impact of working alliance in managing youth anxiety and depression: a scoping review

**DOI:** 10.1038/s44184-023-00021-2

**Published:** 2023-01-30

**Authors:** Jermaine M. Dambi, Webster Mavhu, Rhulani Beji-Chauke, Malinda Kaiyo-Utete, Rhiana Mills, Ruvimbo Shumba, Sidney Muchemwa, Rosemary Musesengwa, Ruth Verhey, Melanie Abas, Colette R. Hirsch, Dixon Chibanda

**Affiliations:** 1 Rehabilitation Sciences Unit, Faculty of Medicine and Health Sciences, University of Zimbabwe, PO Box AV178, Avondale, Harare, Zimbabwe; 2 Friendship Bench, 4 Weale Road, Harare, Zimbabwe; 3 Centre for Sexual Health and HIV/AIDS Research (CeSHHAR), 4 Bath Road, Harare, Zimbabwe; 4 Department of International Public Health, Liverpool School of Tropical Medicine, Liverpool, L3 5QA UK; 5 Mental Health Unit, Faculty of Medicine and Health Sciences, University of Zimbabwe, PO Box A178, Avondale, Harare, Zimbabwe; 6 Department of Psychology, Institute of Psychiatry, Psychology and Neuroscience, King’s College London, London, SE5 8AF UK; 7 Department of Psychiatry, University of Oxford, Oxford, OX3 7JX UK; 8 Health Service and Population Research Department, Institute of Psychiatry, Psychology and Neuroscience, King’s College London, London, SE5 8AF UK; 9 Department of Population Health, Faculty of Epidemiology and Population Health, London School of Hygiene and Tropical Medicine, Keppel Street, London, WC1E 7HT UK

**Keywords:** Psychology, Anxiety, Depression

## Abstract

A working alliance (WA) is a multidimensional construct signifying a collaborative relationship between a client and a therapist. Systematic reviews of therapies to treat depression and anxiety, almost exclusively in adults, show WA is essential across psychotherapies. However, there are critical gaps in our understanding of the importance of WA in low-intensity therapies for young people with depression and anxiety. Here, we describe an initiative to explore the effect of WA on anxiety and depression outcomes in youth aged 14–24 years through a scoping review and stakeholders’ consultations (*N* = 32). We analysed 27 studies; most were done in high-income countries and evaluated one-on-one in-person therapies (18/27). The review shows that optimal WA is associated with improvements in: relationships, self-esteem, positive coping strategies, optimism, treatment adherence, and emotional regulation. Young people with lived experience expressed that: a favourable therapy environment, regular meetings, collaborative goal setting and confidentiality were vital in forming and maintaining a functional WA. For a clinician, setting boundaries, maintaining confidentiality, excellent communication skills, being non-judgmental, and empathy were considered essential for facilitating a functional WA. Overall, a functional WA was recognised as an active ingredient in psychotherapies targeting anxiety and depression in young people aged 14–24. Although more research is needed to understand WA’s influence in managing anxiety and depression in young people, we recommend routine evaluation of WA. Furthermore, there is an urgent need to identify strategies that promote WA in psychotherapies to optimise the treatment of anxiety and depression in young people.

## Background

A working alliance, also referred to as a therapeutic alliance, is a multidimensional construct signifying a collaborative relationship between a client and a therapist^1–3^. A functional Working Alliance (WA) hinges on shared confidence that therapy will be helpful. Also, there is concurrence between the client and therapist over the assignment of therapy tasks; the relationship includes mutual trust and reciprocal liking^1,4^. Collectively, a functional WA has three salient elements, i.e., the creation of a bond between the patient and therapist, agreement in setting therapy goals and guiding scheduled tasks necessary for attaining therapy objectives (goal setting)^1–3^.

Systematic reviews of therapies to treat depression and anxiety, almost exclusively done in adults, demonstrate that WA is essential across psychotherapies^1–7^. A functional WA, as perceived by both client and therapist, predicts greater uptake of interventions, client engagement, adherence to treatment, and symptom reduction^1,5,7^. Conversely, ruptured and/or low WA reduces the effectiveness of known-efficacious treatments^2,4^. Of the available reviews on young people (YP), Sun et al. (2019), looking at cognitive behavioural therapy (CBT) for internalising disorders in YP, found that goal setting, parental involvement, relapse prevention, and booster sessions explained only 14% of the variance predicting treatment outcomes^6^. This implies that other factors, potentially including WA, explain treatment effects. Another meta-analysis in youth demonstrated that a strong WA is predictive of positive treatment outcomes in family-involved treatment for youth problems^4^. However, there are critical gaps in our understanding of the importance of WA in individual psychotherapies and low-intensity therapies (e.g., behavioural activation, psychoeducation, and problem-solving therapy) for YP with depression and anxiety. Also, there is inconclusive evidence regarding the putative mechanism through which WA optimises treatment outcomes^1–7^. Consequently, Welcome Trust has launched Active Ingredients commissions to understand elements essential for the prevention, ongoing treatment and management, and prevention of relapse of anxiety and depression in youth aged 14–24 years^8^.

The Active Ingredients commission seeks to understand the putative mechanisms by which treatments bring about clinical changes, including understanding the context and possible harms^8^. For instance, some teams have demonstrated the usefulness of interventions such as self-compassion, physical activity, emotional regulation, and problem-solving therapy (PST) in managing anxiety and depression in youth^9,10^. Regardless of intervention effectiveness, potential active ingredients such as WA are essential for optimal outcomes^1–7^. For instance, despite PST being effective in managing depression in youth^11^, a poor WA would invariably lead to poor clinical outcomes^1–7^.

Since most mental health problems (75%) initially occur in youth^12^, it is vital that WA is understood in order to maximise the effectiveness of prevention interventions, treatment and ongoing management of anxiety and depression in youth^11^. Understanding the role of WA could also refine the implementation of known effective treatments. More importantly, there have not been previous attempts to appraise evidence on WA in youth with key input from youth with lived experience of anxiety and/or depression. Here, we describe an initiative to explore the effect of WA on anxiety and depression outcomes in youth aged 14–24 years through a scoping review and stakeholders’ consultations.

## Methods

This initiative involved a series of successive, complimentary activities (scoping review, stakeholder consultations and validation workshops), described in detail subsequently. First, we conducted a scoping review, followed by stakeholders’ interviews and went on to summarise and synthesise the findings collaboratively with YP through validation workshops.

### Scoping review

We undertook a scoping review to appraise the evidence of the impact of WA on depression and anxiety in young people aged 14–24 years. The following research questions guided the scoping review:

1. Does better WA improve clinical outcomes of interventions for young persons (14–24 years) with anxiety and depression?
2. What WA elements (bond, goal, and task) influence treatment outcomes?
3. What factors (e.g. patient characteristics, therapy format and delivery mode, etc.) influence the WA-outcome relationship?
4. Can ruptured/dysfunctional working alliances have negative/harmful effects on the clients and or treatment outcomes?

The review was conducted following the PRISMA-ScR checklist^13^ (see Supplementary File 1).

#### Eligibility criteria

The following criteria was applied in selecting articles:

- Study designs/interventions: we included all quantitative designs (randomised controlled trials, cross-sectional, cohort and case-control studies). Systematic reviews, editorials, qualitative studies, case studies and study protocols were excluded.
- Participants/settings: we analysed all studies reporting on WA in young persons with anxiety and/or depression aged 14–24 years across all settings. In cases where the participants’ average age was beyond 24 years, we only included the study if ≥50% of participants were in the 14–24 age range.
- Language: we only analysed articles published in the English language; our preliminary searches did not yield/reveal articles published in languages other than English.

#### Information sources

Peer-reviewed articles were searched/retrieved from these electronic databases; PubMed, CINAHL, Scopus, PsychINFO and Africa-Wide information. Databases were searched from inception through August 2021. Where only an abstract was available online, an attempt to contact the lead author was made, requesting the full article to ensure literature saturation. The article was excluded from the review if there was no response in two weeks following three email reminders. We also reviewed grey literature using the Google Scholar search engine to search potential databases such as university databases and conference proceedings, among others, for articles. For completeness, we also performed both backward and forward searches of the reference lists of identified articles and databases, respectively.

#### Search strategy

As an illustration, articles in CINAHL were searched using the following Boolean logic operators: (“working alliance” OR “therapeutic alliance” OR “collaborative alliance” AND “anxiety OR depression OR anxiety/depression OR (anxiety AND depression)” AND “young people OR young adults OR teenagers OR Adolescent*“.

#### Selection of sources of evidence

First, three early career researchers and three young people with lived experience of anxiety and/or depression piloted the data collection tool by extracting data from five (5) articles. Two researchers then independently searched articles using a pre-defined search strategy (see above). The principal author (JMD) then imported the searches into Mendeley Software and removed duplicates. Afterwards, another set of independent researchers screened the articles by title and abstract. JMD then performed backwards and forward citation searches to identify other potential articles. More senior researchers reviewed the list of identified articles to check for the completeness of the searches.

#### Data charting process

Once searches were finalised, two researchers retrieved the full articles and extracted the data. Two researchers independently cross-examined all extracted data, and disagreements were resolved through discussion with a more experienced researcher who made the final verdict.

#### Data items

The data extraction sheet included information/variables such as author, year, age group, primary and secondary outcome measures, and critical findings. WA, anxiety, and depression were the primary outcomes for this scoping review. Secondary outcomes included variables such as changes in relationships, and coping mechanisms, amongst other relevant outcomes.

#### Synthesis of results

Results were qualitatively synthesised per study objectives. Study outcomes were summarised per study design.

### Key stakeholder consultations

Using semi-structured interviews, we consulted various stakeholders to explore further issues identified through the scoping review. Sampling was purposive to ensure informed discussions and a range of perspectives. Stakeholders therefore comprised: lay health counsellors (*n* = 6), psychologists (*n* = 2), occupational therapists (*n* = 2), psychiatrists (*n* = 2), and YP with lived experiences (*n* = 20). Discussions explored various issues, including WA elements considered most important by both YP with anxiety and/or depression and therapists. We also explored the therapist and YP patient characteristics (e.g., age, gender) that influence the therapeutic alliance-outcome relationship. The outbreak of SARS-CoV-2 coincided with the commencement of data collection activities, which necessitated a shift from in-person to phone interviewing. Discussions were informed by a guide (see Supplementary Files 2 and 3), and text data from the interviews were entered in real-time into hard copy templates. Where possible, verbatim quotes were included. Initially, first-level coding was done by reading the interviews and identifying key emerging issues. The next step involved identifying patterns and linkages within the established codes and collating the codes into thematic areas using thematic analysis^14^.

#### Data synthesis/validation workshops

We convened an initial workshop to triangulate/synthesise findings from the scoping review and stakeholder consultations. We summarised the scoping review and stakeholders’ findings in a simplified manner; YP representatives reviewed this to ensure simplicity and relevance. The validation workshops followed a modified “theory of change” approach to map the “pathway to impact”. Using visuals and placards, we co-created a visual graphic model summarising participants’ views and data interpretation. The output of this initial workshop was the visual first iteration of the mechanistic framework hypothesising pathways by which WA influences treatment outcomes. Subsequently, YP representatives independently consulted with the community advisory group (CAG), constituting YP with lived experiences, the appropriateness of the hypothesised model. Finally, we convened a second workshop to finalise the insight analysis collaboratively with YP.

### Involvement of young persons (YP)

We worked collaboratively with young people with lived experience of anxiety and/or depression, and their specific roles included:

I. Project design: e.g., developing a unified definition of WA, identification/mapping of key stakeholders.
II. Literature searches: YP representatives previously trained and involved in systematic reviews assisted with the pre-application screening of available reviews and assisted with article screening for the actual scoping review.
III. Data collection: e.g., co-facilitating stakeholders’ interviews.
IV. Analysis and synthesis: e.g., reviewing themes emerging from stakeholders’ interviews.
V. Dissemination: e.g., co-developing output animation.

## Results

### Scoping review

#### Study selection

**Fig. 1:**
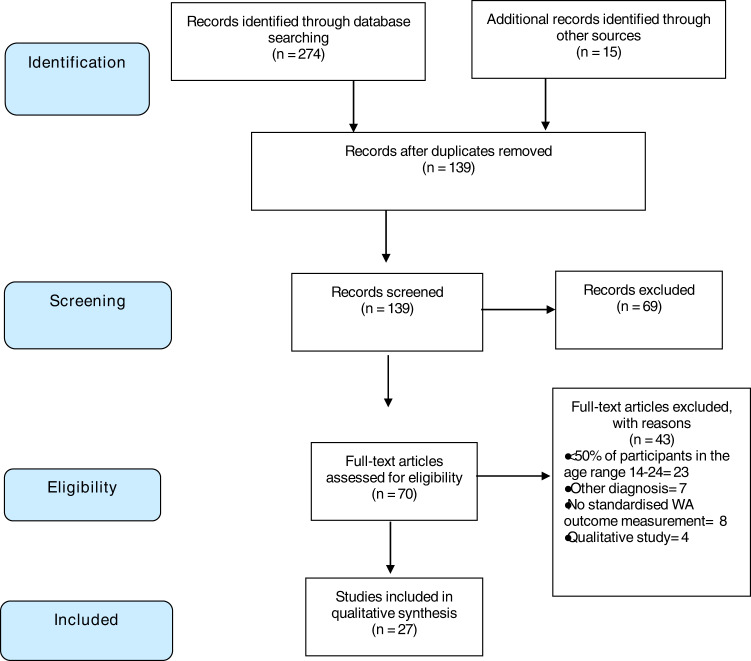
Flowchart of article search and selection process. We identified 274; 139 were duplicates. After applying the selection criterion, 27 articles were analysed for the present review. Adapted from the PRIMA-ScR guidelines.

Initially, 274 articles were identified by searching academic databases and grey literature. After filtering duplicates and screening by title and abstract, 70 full articles were retrieved. Further screening was applied, and 27 articles were included in the qualitative synthesis (see Fig. 1).

#### Study characteristics

Nearly all studies (26/27) were conducted in high-income countries (HICs), mainly in the US (12/27). The Diagnostic and Statistical Manual of Mental Disorders (DSM) was the most used diagnostic tool (10/27). Cognitive behavioural therapy (CBT) was the most commonly applied treatment modality (16/27), and most sessions were done individually (18/27) as opposed to in a group format (5/27). Only two (2) studies utilised digital therapy platforms, with the remainder being in-person therapies (25/27) (Table 1).

**Table 1:** Study characteristics.

First author (year)	Country- income classification	Setting/design	Target population(s)	Age	Male:female ratio (%)	Intervention	Therapy format	Clinical diagnostic tool	Anxiety outcome measure(s)	Depression outcome measure(s)	Anxiety/depression outcome measure	Secondary outcome measure(s)
Feeley et al. (1999)^31^		Facility	Patients with major depressive disorder, *N* = 25	32.9 (SD 11.2)	12:88	CBT		Research Diagnostic Criteria		BDI		CSPRS
Barber et al. (2000)^16^	USA- HIC	Facility	Patients with depression, anxiety, anxiety/depression, personality disorders, *N* = 80	38.4 (SD 11.8); Range 19–66	46:54	Supportive -expressive, dynamic therapy	Individual	Structured Clinical Interview for DSM-III-R	GAD-7	BDI		
Schwartz et al. (2003)^32^	USA- HIC	RCT (12 academic centres)	Adults with major depression *N* = 455	CBASP - 43.7 (SD = 10.6); Combined- 44.6 (SD = 10.5)	CBASP - 37.5%; Comb - 29.3%	CBASP, *N* = 228) or a combination of CBASP and Nefazodone (*N* = 227)	Individual	SCID-II		Hamilton Rating Scale HRSD		LIFE Base
McLeod and Weisz (2005)^27^	USA- HIC	Facility	Children (*N* = 22)	10.3 (SD = 1.8; range 8–14)	40.9:59.1	Child treatment as usual	Individual	DISC	STAIC	CDI		i) CBCL ii) TBQ
Constantino et al. (2010)^38^	Canada- HIC	Facility	Patients with depression *N* = 74	39.9 (SD 11.3)	26:74	IPT	Individual	SCID-IV-TR		BDI-II		IMI
Johansson et al. (2011)^24^	Norway- HIC	RCT	Patients with common mental disorders, *N* = 100	36.9 (SD 9.3)	44:56	Dynamic psychotherapy	individual	DSM-III-R			SCL-90-R	i) PFS ii) GAF iii) IIP-C
Strunk et al. (2012)^21^	USA- HIC	Facilities /RCT	Depressed outpatients, *N* = 176	43.8 (SD 13.0; range 18–80)	42:58	Cognitive therapy				BDI–II		i) CSPRS
Levin et al. (2012)^25^	USA-HIC	RCT	Adolescents with anxiety and depression, *N* = 31	Mean 15.9 (SD 1.7)		Unified Protocol for the Treatment of Emotional Disorders in Youth	Individual	DSM-IV	ADIS Child Version, Parent and Child Reports		Revised Child Anxiety and Depression ScaleRCADS	i) AAQ ii) MSPSS iii) CASAFS
Stubbings et al. (2013)^30^	Australia- HIC	RCT	Patients with mood disorders, *N* = 26	30 (SD 11): range 18–59	42:58	CBT	Individual in-person vs. videoconference	SCID	i) DASS ii) HAQ iii) ASI	BDI-II		i) QLES ii) OCI iii) PSWQ iv) CSQ-8 v) TSQ
Dinger et al. (2013)^39^	USA- HIC	Facility	Patients diagnosed with major depression disorder, *N* = 156	37.5 years (SD 12.1)	29.1:60.9	Psychodynamic therapy		DSM-IV		HRSD		Inventory of Interpersonal ProblemsIIP
Andersson et al. (2012)^40^	Sweden-HIC	RCT	Participants with; i) depression (*n* = 49) ii) generalised anxiety disorder (*n* = 35) iii) social anxiety disorder (*n* = 90).	i) 38.9 (SD 13.5) ii) 40.0 (SD 11.) iii) 37.7 (SD 11.4)	i) 25:75 ii) 19.4:80.6 iii) 41.7:59.3	CBT	Individual; internet-based		i) (PSWQ) ii) LSAS-SR	BDI		
Hersoug et al. (2013)^19^	Norway- HIC	RCT	Patients with depressive, anxiety and personality disorders, *N* = 100	36.9 (SD 9.3) range 21–57	44:56	Psychodynamic psychotherapy	Individual	DSM-III-R (SCID-II).				i) (QOR) ii) Global expectancy iii) Target expectancy iv) (PFS) v) IIP-64
Arnow et al. (2013)^33^	USA- HIC	RCT	Adults with chronic depression, *N* = 224	46.5 (SD 11.3) range 18–75	46.4:53.6	Cognitive Behavioural analysis system of psychotherapy	Individual	DSM–IV–TR (SCID-P)				i) CBASP Therapist Adherence Rating Scale ii) GAF
Lorenzo-Luace (2014)^17^	USA- HIC	Clinical trial	Patients with depression, *N* = 60	40.3 (SD 11.5)	42:58	Cognitive therapy	Individual	Structured Clinical Interview for DSM-IV		i) BDI-II ii) HRSD		NEO-FFI
Chu et al. (2014)^41^	USA- HIC	Outpatient clinic- Longitudinal	i) Youth with common mental disorders, *N* = 69 ii) Parents, *N* = 69	12.5 (SD 2.6)	47.8:52.2	CBT	Individual	DSM–IV–TR	(ADIS-IV- child) child/parent interviews		RCADS –parent version	i) STAIC –parent version ii) CBCL iii) RSQ–parent version
McEvoy et al. (2014)^26^	Australia -HIC	Facility	Patients with unipolar depressive disorder or anxiety disorder, *N* = 199	37.3 years (SD 12.5, Range = 18–73).	30.2:69.8	CBT	Either individual (*n* = 84) or group (*n* = 115)	MINI	BAI	BDI-II	IIP-32	
Webb et al. (2014)^22^	USA- HIC	Psychiatric unit	Patients with major depressive disorder, *N* = 103	36.02 (SD = 13.71, range 18–63)	36:64	CBT	Either individual or group	MINI		CES-D-10		CEQ
Tschschke et al. (2015)^28^	Switzerland- HIC	Facility	*N* = 81	39.6 (SD 11.8, range 17–71	43.2:56.8	8 different psychotherapies	Individual	DSM-IV			GAF	GSI of BSI
Heynen et al. (2017)^37^	Netherlands- HIC	Prospective longitudinal design	Patients with common mental disorders, *N* = 164	38.5 (SD 13.0)	41:34	Arts therapy	Individual	DSM‐IV			BSI	
Lorenzo-Luaces et al. (2017)^34^	Netherlands- HIC	RCT	Data from a RCT comparing CBT and SPSP for patients with depression (*N* = 341; 164 in CBT and 177 in SPSP).	39.49 (SD 10.2)	32:68	CBT and SPSP	Individual	(MINI)		(HRSD)		
Rubel et al. (2017)^15^	Germany- HIC	Outpatient clinic	Patients with various disorders, primarily depression and anxiety *N* = 1550	35.98 (SD 12.70)	36.9:63.1	CBT	Individual	SCID-I			HSCL-11	
Falkenström et al. (2018)^34^	Kenya-LMIC	Outpatient clinic- Observational	Patients with CMDs, *N* = 345	28.9 (SD 9.8)	72.6:27.4	Various psychotherapies	Individual	ICD-10			CORE—Outcome Measure	CORE therapy assessment form
Khalifian et al. (2019)^20^	USA- HIC	Facility/prospective cohort	Patients with depression, anxiety, anxiety/depression, other	33.4 (SD 13.7); Range 18–70	44.6:55.4	CBT	Individual and group	(MINI)	GAD-7	CES-D-10		(BASIS-24)
Doorn et al. (2019)^36^	Canada- HIC	Clinical/naturalistic study	Patients with personality dysfunction, *N* = 80	37.6 (SD 10)	30:70	Group therapy	Group	DSM‐IV		BDI-II		i) RSE ii) QOR
Steinhert et al. (2019)^18^	Germany- HIC	University hospital	Patients with high physical and mental comorbidity, *N* = 709	39.6 (range 17–70)	38.1:61.9	Psychodynamically oriented psychosomatic treatment	Individual and group	ICD-10			HADS	i) SCL-90-) ii) IIP (IIP) iii) PSQ-30
Goldstein et al. (2020)^29^	USA- HIC	Facility/ Non-randomised comparison	i) Therapists - qualified vs. student therapists ii) Patients with major depression disorder	Students - 37.4 (SD 12.6) Qualified 40.3 (SD 11.5)	41:59	Cognitive Therapy	Individual	Structured Clinical Interview for DSM-SCID-IV	BAI	i) HRSD ii) BDI-II		Ways of Responding Scale WOR
Zelencich et al. (2020)^23^	Australia- HIC	RCT	Therapist-client dyads, *N* = 31	47.32 (SD 15.26)	71:29	CBT	Individual		HAD	DASS		i) HRS-II ii) -HAACS

*n* study sample size, *SD* standard deviation, *BDI* Beck’s Depression Inventory, *CSPRS* Collaborative Study Psychotherapy Rating Scale, *HIC* High income country, *DSM-III-R* Diagnostic and Statistical Manual of Mental Disorders-III—Revised, *GAD-7* General Anxiety Disorder-7, RCT - randomised controlled trial, *SCID-II* The Structured Clinical Interview for DSM-IV Axis II Personality Disorders, LIFE Base Longitudinal Interval Follow-Up Evaluation Base, *CBASP* Cognitive–Behavioural Analysis System of Psychotherapy, *DISC* Diagnostic Interview Schedule for Children, *STAIC* State-Trait Anxiety Inventory for Children, *CDI* Children’s Depression Inventory, *CBCL* Child Behaviour Checklist, *TBQ* Therapist Background Questionnaire, *SCID-IV-TR* Structured Clinical Interview for DSM-IV (clinician administered), *BDI* Beck’s Depression Inventory II, DSM-III-R -, SCL-90-R, PFS Psychodynamic Functioning Scales, *GAF* Global Assessment of Functioning, *IIP-C* Inventory of Interpersonal Problems – Circumplex version, *CSPRS* Collaborative Study Psychotherapy Rating Scale, *CBT* Cognitive behavioural therapy, *RCADS* Revised Child Anxiety and Depression Scale, *ADIS-Child* Anxiety Disorders Interview Schedule-Child, *AAQ* Adolescent Attachment Questionnaire, *MSPSS* Multidimensional Scale of Perceived Social Support, *CASAFS* Child and Adolescent Social and Adaptive Functioning Scale, *DASS* The Depression Anxiety and Stress Scale, *HAQ* Health Anxiety Questionnaire, *ASI* Anxiety Sensitivity Index, *QLES* The Quality-of-Life Enjoyment and Satisfaction scale, *PSWQ* Penn State Worry Questionnaire, *OCI* Obsessive-Compulsive Inventory, *CSQ-8* Client Satisfaction Questionnaire, *TSQ* Telehealth Satisfaction Questionnaire, *IIP* Inventory of Interpersonal Problems, *HRSD* Hamilton Rating Scale for Depression, *LSAS-SR* - Liebowitz Social Anxiety Scale self-report version, *QOR* Quality of Object Relations Scale, PFS Psychodynamic Functioning Scales, *GAF* Global Assessment of Functioning Scale, *NEO-FFI* NEO Five-Factor Inventory, *RSQ* Responses to stress questionnaire, *BAI* Beck anxiety inventory, MINI Mini International Neuropsychiatric Interview, *CEQ* Credibility Expectancy Questionnaire, *CES-D-10* Center for the Epidemiological Studies of Depression-10, *GSI* Global Severity Index, *BSI* Brief Symptom Inventory, *SPSP* Short Psychodynamic Supportive Psychotherapy, *HSCL-11* Hopkins-Symptom-Checklist, *CORE* Clinical Outcomes in Routine Evaluation, *BASIS-24* The Behaviour and Symptom Identification Scale, *RSE* Rosenberg Self‐Esteem Scale, *SCL-90-R* Symptom Checklist-90-Revised, *PSQ-30* Perceived Stress Questionnaire, *HADS* Hospital Anxiety and Depression Scale, *ICD-10* International Classification of Diseases, *WOR* Ways of Responding Scale, *HAACS* Homework Adherence and Competence Scale, *IMI* Impact Message Inventory, *IPT* interpersonal therapy.

#### Working alliance measurement and association with key clinical outcomes

Table 2 outlines the assessment timing, assessor, key findings, and overall synthesis per study. The working alliance inventory was the most applied outcome measure (12/27). WA was mainly measured at the beginning (14/27) or at both the beginning and end of therapy (17/27). Most assessments were completed by clients (15/27), with therapists’ ratings only recorded in two studies. A functional WA was recognised as an active ingredient in psychotherapies targeting anxiety and depression in young persons aged 14–24 years. Most studies (24/27) revealed a positive association between WA and clinical outcomes. Two studies revealed a null association, with a single study showing a negative association.

**Table 2:** Working alliance outcomes.

Author (year)	Outcome measure(s)	WA assessment timing			WA assessor			Main findings	Main findings 2	Main findings 3	Main findings 4	Main findings 5	Association
		Early	Mid	Late	Client	Therapist	Observer						
Feeley et al. (1999)^31^	Penn Helping Alliance rating scalePHAS	Yes		Yes			Yes	Measures of in-session therapist behaviour and therapist-patient interactions were correlated with prior and subsequent symptom change	“Concrete” subset of theory-specified therapist actions, measured early in treatment, predicted subsequent change in depression	The therapeutic alliance was predicted by prior symptom change in 1 of the 2 later assessments, but only at a trend level			Positive
Barber et al. (2000)^16^	CALPAS	Yes	Yes	Yes	Yes			Greater WA associated with greater changes in depression scores	Greater decrease in depressive symptoms from intake to the time alliance is assessed is associated with higher alliance level, albeit not very early in treatment				Positive
Schwartz et al. (2003)^32^	WAI-SR	Yes		Yes	Yes			Early alliance significantly predicted subsequent improvement in depressive symptoms after controlling for prior improvement and 8 prognostically relevant patient characteristics.	Neither early level nor change in symptoms predicted the subsequent level or course of the alliance.	Patients receiving combination treatment reported stronger alliances with their psychotherapists than patients receiving CBASP alone			Positive
McLeod and Weisz (2005)^27^	TASC	Yes		Yes	Yes		Yes	Child–therapist alliance during treatment predicted reduced anxiety symptoms at the end of treatment.	Parent–therapist alliance during treatment predicted reduced internalising, anxiety, and depression symptoms at the end of treatment.				Positive
Constantino et al. (2010)^38^	WAI				Yes	Yes		Increased alliance associate with decreased depression					Positive
Johansson et al. (2011)^24^	i) WAI ii) HUS	Yes	Yes	Yes	Yes	Yes		Patient ratings, but not the therapist rating of alliance mediated the association between global expectancy and clinician-rated outcome	Global optimism was associated with patient-rated alliance after first session; the effects waned off with subsequent sessions				Positive
Andersson et al. (2012)^40^	WAI				Yes			Subsample I – both groups improved on the BDI with within group effect sizes being *d* = 2.18 and *d* = 1.39 for the email therapy and guided self-help groups, respectively	Subsample II – the treatment group improved, and for the group who completed the short WAI the within group effect size on the PSWQ was *d* = 1.17	Subsample III – the treatment group improved with a within-group effect size of *d* = 0.97 on the LSAS-SR.			Null
Strunk et al. (2012)^21^	WAI	Yes	Yes	Yes			Yes	Both adherence to Behavioural Methods/Homework and the therapeutic alliance significantly predicted session-to-session symptom change.					Positive
		Early	Mid	Late	Client	Therapist	Observer						
Levin et al. (2012)^25^	i) VTAS ii) WAI	Yes	Yes		Yes	Yes	Yes	Adolescents who indicated greater anxiety and depressive symptoms were rated as having stronger early alliances by independent observers	Strong correlation between WA assessments				
Stubbings et al. (2013)^30^	WAI-S			Yes	Yes			No differences in WA ratings across groups (in-person vs. videoconferencing)					Null
Dinger et al. (2013)^39^	CALPAS	Yes	Yes	Yes			Yes	Interpersonal problems related to communion predicted better alliances, but slower symptomatic improvement	Lower interpersonal distress was associated with an increased likelihood to terminate treatment prematurely				Positive
Hersoug et al. (2013)^19^	WAI	Yes	Yes	Yes	Yes			The alliance alone had a significant impact on long-term on quality of relationships, insight, problem solving and interpersonal problems	Patient characteristic had stronger effect on long-term outcome, over and above the effect of alliance				Positive
Arnow et al. (2013)^33^	WAI-S	Yes	Yes	Yes		Yes		A more positive early working alliance was associated with lower subsequent symptom ratings	The interaction between alliance and psychotherapy type was significant, such that alliance quality was more strongly associated with symptom ratings among those in the CBASP				Positive
Lorenzo-Luace (2014)^17^	WAI–O	Yes					Yes	Greater WA associated with greater changes in depression scores	Participants with <3 prior depression episodes had greater alliance-outcome correlation				Positive
Chu et al. (2014)^41^	Therapeutic alliance scale for children/adolescents	Yes	Yes	Yes	Yes	Yes		Pre-treatment anxiety predicted initial alliance scores; depression symptoms and engagement coping style predicted postexposure slope; and no variables predicted preexposure growth	Depressive symptoms predicted less linear growth and engagement coping predicted greater growth during exposure session	Therapist-reported alliance ratings may grow over the course of manual-based CBT, even during exposure-focused sessions			Positive
McEvoy et al. (2014)^26^	HAQ-II	Yes		Yes	Yes			For those receiving individual therapy, those with a stronger early therapeutic alliance were more likely to complete treatment, whereas symptom severity and pre-treatment interpersonal problems were not significantly related to attrition	For those receiving group therapy, the strength of early therapeutic alliance was unrelated to attrition, whereas those with more severe depression and anxiety symptoms and more severe pre-treatment interpersonal problems were more likely to discontinue treatment	For those receiving group therapy pre-existing interpersonal problems were associated with higher attrition and poorer outcomes for treatment completers	For those receiving individual therapy, stronger therapeutic alliance was associated with treatment completion but not with superior outcomes in terms of depression or anxiety symptoms		Positive
		Early	Mid	Late	Self-report	Therapist	Observer						
Webb et al. (2014)^22^	WAI-S		Yes	Yes	Yes			Alliance and treatment outcome expectancies significantly predicted subsequent depressive symptom change	The alliance was significantly associated with prior symptom improvement				Positive
Tschschke et al. (2015)^28^	HAQ	Yes		Yes			Yes	Different types of psychotherapy differ significantly in their degree of treatment adherence Therapeutic alliance was not directly correlated with treatment outcome	There was no statistically significant association between the type of psychotherapy and its outcome, or between the degree of therapists’ treatment fidelity and the treatment outcome	There were significant associations between therapists’ degree of professional experience, clients’ initial psychological burden, and treatment response	Clients’ severity of psychological problems prior to treatment predicted quality of therapeutic alliance	Therapists’ treatment adherence was predicted by therapists’ professional experience and by the quality of the therapeutic alliance	Negative
Heynen et al. (2017)^37^	WAI-12		Yes		Yes			High alliance was overall associated with decreased depression and anxiety	Increase in WA over time				Positive
Lorenzo-Luaces et al. (2017)	Helping Alliance Questionnaire HAQ			Yes	Yes			The alliance was a predictor of symptom change (*d* = 0.33)					Positive
Rubel et al. (2017)^15^	SR	Yes	Yes	Yes	Yes			Better session-specific coping skills, better therapeutic alliance, and deeper emotional involvement were followed by next session symptom improvements	Coping skills were especially helpful when combined with a better therapeutic relationship quality				Positive
Falkenström et al. (2018)^34^	Session Alliance Inventory SAI	Yes		Yes	Yes			Changes in the working alliance from session to session predicted reduction in psychological distress symptoms at the following session					Positive
Khalifian et al. (2019)^20^	WAI-S	Yes		Yes	Yes			Improvement in the alliance was associated with improved post-treatment relationship functioning	Patients who experienced an improvement in therapeutic bond and tasks reported less relationship difficulties at post-treatment	Improvement in goals was significantly related to lower relationship difficulties at post-treatment for patients with higher relationship difficulties at pre-treatment			Positive
Doorn et al. (2019)^36^	Edmonton Therapeutic Alliance Scale			Yes	Yes			High alliance was overall associated with a change increased self-esteem and decreased depression					Positive
Steinhert et al. (2019)^18^	HAQ	Yes		Yes	Yes	Yes		For patients responding to treatment a significantly better helping alliance was found, corresponding to a large effect					Positive
		Early	Mid	Late	Self-report	Therapist	Observer						
Goldstein et al. (2020)^29^	WAI-S	Yes		Yes	Yes			WA was superior for clients treated by students than qualified therapists	Both alliance and skill acquisition were moderately correlated with therapeutic gains in changes in depression scores				Positive
Zelencich et al. (2020)^23^	WAI-SR-O	Yes					Yes	Higher levels of therapist competence in reviewing homework were associated with greater improvement in anxiety and/or depression symptoms					Positive

*WA* Working Alliance, *PHAS* Penn Helping Alliance rating scale, *CALPAS* California Psychotherapy Alliance Scale, *WAI* Working Alliance Inventory, *WAI-SR* Working Alliance-Self reported, *HUS* Help and Understanding Scale, *CBASP* Cognitive–Behavioural Analysis System of Psychotherapy, *TASC* Therapeutic Alliance Scale for Children, *VTAS* Vanderbilt Therapeutic Alliance Scale, *WAI-O* Working Alliance Inventory–Short Observer-Rated version, *HAQ-II* Helping Alliance Questionnaire 2, *WAI-S* Working Alliance Inventory short version, *WAI-12* Working Alliance Inventory-12, *SR* Session Report, *SAI* Session Alliance Inventory, *ETAS* Edmonton Therapeutic Alliance Scale, Helping Alliance Questionnaire (HAQ), *CBT* Cognitive Behavioural Therapy, *BDI* Beck’s Depression Inventory, *PSWQ* Penn State Worry Questionnaire, *LSAS-SR* Liebowitz Social Anxiety Scale-Self Report, *ETAS* Edmonton Therapeutic Alliance Scale.

### Stakeholders’ consultations

#### Young persons experiencing anxiety and depression views

Consultations with young people indicated what was necessary for developing a WA. A conducive environment, regular engagements, and confidentiality were vital for developing the client-therapist bond. Involvement in setting treatment goals was essential in forming a WA. One respondent said, *“…if two people can agree on the goals and outcomes of the entire session and what they will be working on, l think it will help develop a better connection and bond.”* There was an agreement between the young people with lived experience that involvement in coming up with tasks influenced WA formation, which subsequently affected treatment outcomes. Young people valued being involved in the planning of therapy tasks. Client characteristics such as willingness to engage in therapy, politeness, and being expressive were essential for developing a WA. A trustworthy therapist with good communication skills was considered ideal for developing a WA. Also, cultural background, inappropriate dressing, therapists’ age, gender, and religion were potential barriers to developing working alliances. Patients much-preferred therapists of the same gender. A female client stated, “*…my counsellor was female, and it helped me a lot.”* Also, respondents preferred more experienced counsellors and therapists and those of similar religious orientations (see Table 3).

**Table 3:** Young person’s views.

Domain	Subtheme	Verbatim quote(s)
Definition of WA	A mutual co-operation between a patient and counsellor	*“Is the ability to connect and agree and work together with your counsellor” (Respondent D2)*
*WA elements:* Bond	Conducive environment	*“Privacy is very important. What happened when we were having our counselling sessions there was not much space so l could hear what the other counsellor and client were discussing which is not good, l would not want that.” (Respondent D3)*
	Regular engagements	*“I think keeping in touch and meeting face to face, even monthly.” (Respondent V1)* *“Regular communication…” (Respondent V3)*
	Confidentiality	*“At first, when we started sessions when I saw counsellor talking to someone else, I felt insecure thinking that confidentiality is at stake, but after reading the consent forms, I had a better understanding and was assured of confidentiality.” (Respondent R2)*
Goals	Decision on treatment goals	*“When we had those sessions, l was the one who made all the decisions. I was involved one hundred percent. Because after asking the questionnaire, the counsellor would then ask me, ‘How do you think we can help each other.’ That’s when l will start setting the goals l need and what l expect to accomplish for my problem to be solved.” (Respondent D3)*
	Involvement in treatment goals’ influence on outcome	*“I think to a greater extent. What happens is before l start receiving counselling l will be having these crazy decisions about the problem l will be facing at that time, but when you have received counselling services you are actually able to make better decisions for yourself.” (Respondent D3)* *“Okay firstly l can say, to a greater extent, if two people can agree on the goals and outcomes of the entire session and what they will be working on, l think it will help develop a better connection and bond.” (Respondent D1)*
Tasks	Client involvement in tasks	*“Counsellor would give me support on the task that l suggested, not making the decisions for me. I was greatly involved.” (Respondent R1)*
	Task’s influence on treatment outcome	*“It is to a larger extent; I would do homework or research pertaining my problem then comeback with the feedback. We received the feedback from the homework together and came up with the solutions.” (Respondent T1)*
	Task’s influence in development of WA	*“…it’s just to a greater extent because it gives the client the room to express, how they feel because they will have had time to think about it after the sessions so they can have better ways to express their feelings and develop that bond with the counsellor.” (Respondent D1)*
Key elements required for the developing a functional WA	Willingness	*“…client should be committed, time conscious because coming late for sessions would mean you won’t access counselling services fully…” (Respondent R1)*
	Good manners	*“I think being polite, like we know you have issues but be polite, sometimes someone goes through something that they get angry, but if you speak in a way that the right people would be able to help you out.” (Respondent C2)* *“First one should be responsible, have good manners, they should greet.” (Respondent C3)*
	Expressive/able to open up	*“I think client should be a person who is able to share their problems with the counsellor…” (Respondent V2)* *“As a client you must be willing to participate in the counselling sessions you must not hold anything back for anything.” (Respondent D3)*
	Trustworthiness	*“Therapist should be a trustworthy person…” (Respondent V2)* *“The therapist also has to be friendly, and what else, they have to be trustworthy…” (Respondent R2)*
	Communication skills	*“Therapist should have good communication skills…” (Respondent T1)* *“Speaking in a polite manner should greet, should do his duties.” (Respondent C3)*
	Counselling skills	*“Therapist should be a trustworthy person, a good listener and reliable as well.” (Participant V2)* *“The therapist has to be patient…” (Participant D2)*
Influence of culture on WA	Cultural background	*“There could come a discussion about God; hmm, let’s say the other one is Islamic and the Christian, so there may be disputes on that”. (Respondent B1)* *“The therapist has to be diverse; he or she has to know what happens in other cultures to be able to create a working alliance with any client that he or she is talking to other than his own culture.” (Respondent D2)*
	Dress code	*“Dressing and character affect working alliance negatively. Take for instance if the therapist is not wearing professional dressing it could be disruptive”. (Respondent T1)*
	Communication patterns	*“Yah, I think if people are coming from different cultures, it can affect how they work together. Here l am talking about communication patterns, language… These can become major barriers to having that bond with my counsellor, and the counsellor with me as well.” (Respondent D1)*
Influence of therapist’s socio-demographics’ on WA	Age	*“I think it affects because if someone is young like me, they can help me as they sometimes face what I am going through, however at the same time l feel there are other problems they can’t help me with because of lack of experience as they are still young.” (Respondent V3)*
	Gender	“…*if the counsellor is a man, gender becomes a barrier.” (Respondent V3)* *“…my counsellor was a female, and it helped me a lot.” (Respondent C3)* *“Yes, it can be changed; maybe the client may choose the gender that they feel they are comfortable with during counselling sessions.” (Respondent R2)*
	Religion	*“Religion can be a barrier as we come from different religions, for example, if l am Muslim, yet they are Christian…” (Respondent V3)* *“Uhm, the religion, I do not think it affects anything.” (Respondent D3)*

#### Clinicians’ views on working alliance

The conceptualisation of working/therapeutic alliance aligned with the working definition. However, some lay counsellors were unsure of the term’s meaning. After further probing using descriptors and examples, the lay counsellors could relate to the concept. Most clinicians viewed personal connection with a client as an essential aspect of improving treatment outcomes that facilitates understanding between the client and clinician, builds trust, facilitates empathy, and enables the application of good listening skills. Setting boundaries, maintaining confidentiality, and being non-judgmental and empathetic were considered pre-requisite clinician attributes for forming a WA. Also, goal setting was deemed integral to successful therapy. Furthermore, the importance of participants’ motivation levels, mutual agreement on goals, and the number of times the goals were set and reviewed were considered crucial for forming and maintaining a WA (see Table 4).

**Table 4:** Clinician’s views on WA.

Domain	Subtheme	Verbatim quotes
Definition of WA	Cooperative working relationship between client and clinician	*“Relationship that they (client and clinician) have, that is directed to achieve certain goals”* (Lay counsellor C1) *“An agreement between the client and a clinician/counsellor with regards to confidence and setting of goals in the session”* (Lay counsellor B1)
	An unfamiliar term	*“I have not yet heard about the term Working Alliance”* (Lay counsellor T1) *“It refers to mental health.”* (Lay counsellor T2) *“Maybe you can just give me a definition of the context you are meaning by the term working alliance so that I can answer accordingly.”* (Lay counsellor R2)
Importance of WA	Facilitates understanding between the client and clinician	*“So, if a client and a therapist have a relationship, if there is trust and if there is understanding during the sessions or helps with the outcomes of the session. But if the client does not trust the therapist, it means they are not so free to say whatever er they want to say; it also means they are not free to say whatever they want to say, it also means they cannot express themselves”* (Lay counsellor C1) *“Helps the client to clearly understand the session and to be open when they are having sessions which are very important for the treatment outcome.”* (Lay counsellor T2)
*WA elements:* Bond	Conducive environment	*“It is the counsellor’s responsibility to make sure that the atmosphere is conducive for therapy…”* (Lay counsellor C1)
	Regular engagements	“The bond with clients semes to get stronger with increased number of sessions” (Lay counsellor R2)
Goals	Giving client autonomy	*“I always make sure that the client is in control of the treatment program….especially for the home assignments*…” (Lay counsellor T2)
Tasks	Client involvement in tasks	*“I always make sure to involve the client in the treatment process.. that is what my supervisor always emphasizes”* (Lay counsellor B1) *“You can never go wrong if you involve the client in setting tasks”* (Lay counsellor V1)
	Task’s influence on treatment outcome	“*There is no way you can get any meaningful results if you don’t involve the patient…it is very important to involve them all the way…”* (Lay counsellor R1)
	Builds trust/confidentiality between client and clinician	*“But if the client does not trust the therapist, it means they are not so free to say whatever er they want to say; it also means they are not free to say whatever they want to say, it also means they cannot express themselves,”* (Lay counsellor R1)
	Brings out essential therapist’s skills such as empathy and good listening skills considering cultural values as well.	*“Ummh…you can have a personal relationship where ethics are accommodated, for example, using active and good listening skills to add for a personal connection and empathy, using empathy where there is need for empathy, you know, trust in the end of the counsellor being able to address their ethics realising that the ethics you have been doing are relationship becoming more than it should be then it can actually be addressed.”* Lay counsellor R2)
Key elements required for the developing a functional WA	Goal, bond, and task	*“Goal setting, bond and task, I think these are the key elements which are important”* (Lay counsellor B1)
	Boundary setting	*“…and then l think about it, one of the most important things when going back to the issue of personal connection is boundary. Are we able to set boundaries, within that phase,”* (Lay counsellor V1)
	Confidentiality	*“Okay, so this is informing the client of confidentiality, informing the client of being able to speak openly without being judged and informing the client that information shall not be shared unless they are at the risk of harming themselves or myself in the counselling process.”* (Lay counsellor R2)
	Non-judgmental	*“If there could be that custom that clients are free to express themselves freely, clients should know that everything done by the therapist is to add confidence and to make the client feel safe then the counsellor is also non-judgmental and lets the client express themselves freely in a comfortable way so that we won’t have clients ending up lying or pretending to appear nice.”* (Lay counsellor R3)
	Empathy	*“I think other key components can be empathy, yes empathy can also contribute to the therapeutic alliance because a client will be feeling that you are with him or her.”* (Lay counsellor R1)

### Synthesis of scoping review and stakeholders’ consultations

**Fig. 2:**
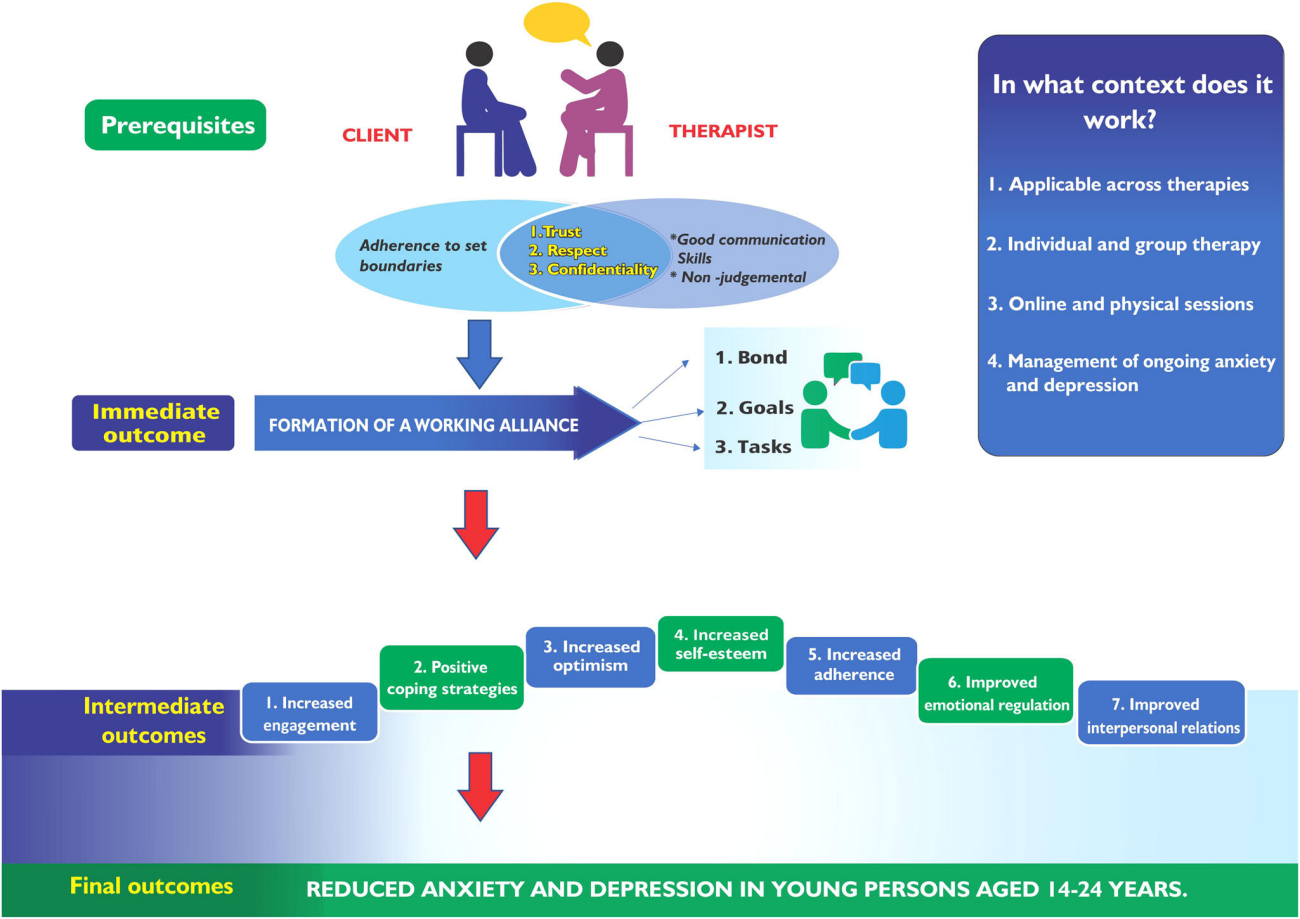
Hypothesised working alliance-outcome relationship. The figure identifies potential mechanisms whereby WA can optimise treatment outcomes for anxiety and depression in young people. © G Manyofa, 2022.

Figure 2 identifies potential mechanisms by which WA can optimise treatment outcomes for anxiety and depression in young people. Bonding between a client and therapist and consensus on therapy goals and tasks are fundamental in developing a WA. Certain conditions, including therapists’ characteristics (e.g., communication skills, empathy), less severe baseline symptoms, initial symptoms change, and regular engagement between the therapist and client, are essential for developing an optimal WA. Once created, a WA can lead to increased adherence to treatment, improved self-esteem, increased treatment satisfaction, and improved relationships and coping skills. Ultimately, WA is associated with increased treatment effectiveness, thus mitigating the burden of anxiety and depression in young persons. WA can potentially optimise treatment outcomes across psychotherapies and treatment formats, i.e., individual vs group therapy and in both physical and digital treatment formats.

## Discussion

Triangulating findings from the scoping review, stakeholder consultations and synthesis workshops, we conclude that WA is an essential active ingredient in psychotherapies addressing anxiety and depression in young persons. Overall, a greater WA is associated with improvements in depression and anxiety in young people. Our scoping review findings are consistent with what has been observed in reviews focusing exclusively on adults^1,2,5,15^.

Despite the universal agreement on the importance of a functional WA, the exact mechanism by which a WA optimises reductions in anxiety and depression remains largely obscure^3^. From literature^1,2,5,16–18^ and our stakeholders’ consultations, it appears that improvements in the WA are associated with improvements in interpersonal relationships, self-esteem, positive coping strategies, optimism, adherence to treatment protocols, and emotional regulation. Improvements in negative psychosocial indices and treatment processes/factors attenuate anxiety and depression symptoms. Consequently, improved mental health can positively influence WA creating a positive feedback loop^1,2,5^. However, some studies included in the scoping review did not find an association between WA and depression or anxiety^2^.

Recent research into psychotherapy with adults has attempted to use advanced statistical modelling techniques to understand the WA-clinical outcome relationship^1^. However, heterogeneity in methodologies makes it difficult to conceptualise and model the association between WA and anxiety or depression^1,2,5^. For instance, in a systematic review by Baier et al. (2020), most studies retrieved demonstrated that WA is a mediator of change. However, reverse causality and mediation by a third confounding variable could not be ruled out^1,5^. Despite the inconsistencies in the literature, it is, thus, vital to explore the most salient element associated with improved WA.

In the scoping review, improvements in bond and tasks were associated with improvements in interpersonal functioning^19,20^. Common mental disorders (e.g. anxiety, depression) are sometimes associated with relationship problems; therefore, establishing a bond with the therapists can affect the client’s social life^21^. It is generally suggested that early WA is essential in the early stages of the therapeutic process. Early bonding between the client-therapist is likely to improve the client’s agreement on the task and subsequent adherence to treatment procedures, ultimately improving treatment outcomes^1^.

The bond formation and task completion depend on patient and therapist characteristics. However, despite forming a WA during sessions, if clients are not provided with coping skills, the mediation of WA in symptomatic relief is attenuated^2,15^. Therapists corroborated this proposition during our stakeholders’ consultations. Elsewhere, in a naturalistic study, Webb et al. (2014) demonstrated that the task component was statistically associated with treatment outcomes after controlling for temporal confounders (patient expectations and prior symptom change)^22^. The same study demonstrated the importance of agreement on concrete tasks as being fundamental to changes in depression in CBT^22^. The importance of task completion is further supported by Zelenchich et al.^23^, who explored the effect of WA on anxiety and depression in youth with acquired brain injury. Facilitation by the therapists in completing tasks was linked to improved functioning and lower anxiety and depression^23^. This is further supported by a systematic review of adult patients undergoing CBT for anxiety disorders; task agreement was more predictive of the WA therapeutic-outcome association, with bond/goals-outcome association equivocal^2^.

We also set out to explore factors “mediators” to the WA-outcome association. Understanding these factors is essential to fostering a functional WA. The following variables can potentially affect the WA-outcome association: client and therapist characteristics, therapist professional experience, the timing of WA formation, therapy type(s), therapy delivery mode, therapy format ((digital/physical and individual/group), and setting (inpatient/outpatient). These are discussed in the subsequent paragraphs.

Pre-treatment patient characteristics, including motivation, hope for change, and expectancy in therapy effectiveness, are precursors to forming a functional WA^19^. For instance, patient optimism towards the potential for improved symptoms is linked to better WA and more favourable treatment outcomes. Consequently, therapists must build realistic expectations and optimism that therapy will be effective^2,22,24^. Also, patients with good attachment histories, adaptive attachment styles and developed social skills are more likely to forge good relationships with therapists, thus improving WA^7^. More critically, positive relations, characterised by an ability to develop a stronger bond between a patient and a therapist, are essential. It creates trust and safety, which spills over to the agreement of goals and subsequent completion of agreed tasks^2^.

Conversely, other patient characteristics (e.g. attachment styles, diagnosis) are potentially detrimental to the WA-outcome relationship^2,21,25,26^. Some studies suggest that a functional WA does not seem to optimise treatment in patients with personality disorders and relationship problems^21,25^. Patients with relationship problems may have challenges connecting with the therapist, which may cause a poor WA, and subsequent poor treatment outcomes^26^. Also, clients with greater relationship difficulties are likely to be more dependent on their therapist; this may lead to challenges in developing their problem-solving capabilities, in turn influencing the ability to forge a functional WA and treatment outcomes^19,26^. Inversely, the greater reliance on the therapist may cause an improved alliance, specifically the client and therapist bond^2^. Furthermore, patients with fewer relationship difficulties may be more realistic in treatment outcomes as they appreciate the difficulty in attaining meaningful change^19^.

Overall, there seems to be no consensus regarding the salient patient characteristics influencing the WA-outcome association. Some studies have concluded that a functional WA does not seem to play a huge role in treatment success; instead, other non-specific factors (e.g. adherence, symptoms severity) seem to influence treatment effectiveness^19^. Therefore, additional research is needed to explore contexts where WA can be harmful or circumstances under which a functional WA can deter patients’ functional recovery. Optimising WA is essential as ruptures in WA can lead to decreased treatment expectancy, which may negatively affect adherence, thus ultimately reducing treatment effectiveness. However, the evidence concerning this is limited, and more research is needed for definitive conclusions^2^.

Our stakeholder consultations identified empathy from the therapist as an essential element across patients. Young people described how providers’ courteous treatment increased their confidence in the treatment process. In Cognitive behavioural therapy (CBT), enhancing a client’s ability to agree on tasks and assignments, including eliciting emotional engagement during therapy, is essential for forging a functional WA. However, evidence from a systematic review exploring the active ingredients of CBT in adult anxiety disorders produced mixed results^2^. Studies are needed to better understand critical windows for good WA to impact therapy outcomes and any moderating effects on the empathy-outcome association^2^. The exploration is essential given that a De Re et al. (2012) meta-analysis demonstrated that therapists’ characteristics hugely contribute to the WA formation regardless of patient diagnosis, research design, and WA measurement^7^. For instance, the therapist’s professional experience seemingly predicts WA cultivation.

From the studies gleaned from the scoping review^22,27,28^ and our consultations, it appears that greater professional experience is associated with better treatment outcomes and greater WA. This is inconsistent with outcomes from a study by Goldstein et al.^29^. exploring the comparability of anxiety/depression symptoms change, skills acquisition and WA between experienced and student therapists. In their study, WA was superior for clients treated by students than qualified therapists. Also, a study showed that videoconferencing was equally effective in treating anxiety and depression; a doctoral trainee psychologist was the therapist^30^. Both alliance and skill acquisition were moderately correlated with therapeutic gains in changes in depression scores^30^. However, our stakeholders’ consultations were indeterminant; clients revealed that age was a potential determinant for establishing WA, with young people preferring to be treated by a similarly aged lay counsellor. A similar-age counsellor was deemed likely to have the same experiences and relate more to a young person experiencing anxiety and depression. Other clients preferred to be seen by a more mature counsellor who could have more experience addressing the issues at hand. In problem-solving therapy and CBT, more experience appears helpful when the patient is still opening up; an experienced counsellor can use their clinical expertise to facilitate problem-solving in the client^15,22,27,28^.

Evidence is inconclusive regarding the requisite timing of WA on changes in mental health functioning^15^. For example, some studies in the scoping review suggested that a more favourable early WA is associated with more significant symptom improvement^3,5,15,17,19,22,25,31–34^, with others reporting a null association^2,16^. The discrepancies have been attributed to differences in outcome measures, the timing of WA assessment and methodological differences, i.e., sample sizes, heterogeneity in study participants, and study designs, amongst other methodological issues^2,4,5,22,34^.

A meta-analysis exploring the WA therapeutic-outcome relationship in CBT for adults with depression revealed that early WA-outcome correlations are marginally lower than mid-and late assessments^5^, thus the need for an early establishment of a WA to optimise treatment outcomes^5^. Another meta-analysis also identified a reciprocal relationship between WA and symptom reduction early in therapy^3^. Early WA was predictive of post-treatment outcomes and optimised drop rates; this association was evident irrespective of baseline symptom severity and was optimised by greater levels of patients’ engagement with treatment and treatment acceptance in the early stages of therapy^3^. However, few long-term studies have assessed the temporal WA therapeutic-outcome association.

WA has been mainly studied using cross-sectional or clinical trials with short follow-ups^5,16^. Hersoug et al. (2013)^19^ explored the long-term effects of WA on 100 patients three years after receiving dynamic psychotherapy for anxiety, depression and personality disorders. This study showed that a functional WA was predictive of long-term changes in mental health outcomes. Furthermore, higher treatment expectations, less severe symptoms, and the ability to create mutually fulfilling relationships with others were predictive of a better functional WA^19^. Of note, the temporal relationship between WA and treatment outcomes is not without controversy^4,22,34^.

A functional WA is considered active across psychotherapies^5,34,35^. Tschschke et al.^28^. demonstrated that WA was essential in predicting clinical outcomes for behavioural, cognitive-behavioural, person-centred, and psychodynamic therapies. This proposition is further supported by systematic reviews and meta-analyses exploring the effects of WA on depression and anxiety for CBT^1–7^. Most studies analysed in the scoping review primarily focused on the relationship between WA and treatment outcomes for standalone psychotherapies. For example, Stunk et al. (2012) explored the relationship between WA, adherence and symptom change in 176 randomised clients receiving combined cognitive therapy and antidepressants for depression in the US^21^. A positive WA was associated with symptom change early in therapy. Furthermore, only the task sub-scale was associated with symptom change. However, multivariate analyses showed that only the task subscale remained the statistically significant predictor after controlling for therapist skill and adherence to cognitive therapy. Taken together, the study showed the importance of agreeing on goals and homework provision to influence both WA and subsequent symptomatic changes in combined therapy^21^. Given the importance of WA across psychotherapies, it is essential to explore the context, i.e. delivery mode, format and settings that influence the WA-outcome association.

In the scoping review, the association between WA and anxiety/depression was the same across delivery modes, i.e., physical vs online therapy and individual vs group therapy^16,35^. Evidence of the WA across physical formats is unequivocal in suggesting that a functional WA optimises in-person therapy outcomes, with evidence across digital platforms still evolving^1,2,4–6^. A pilot randomised controlled trial (*N* = 26) showed that videoconferencing clinically equalled in-person CBT, with client satisfaction and client- and therapist-rated WA comparable across the two groups^30^. Andersson et al.^35^ explored the association between WA and treatment outcomes in guided CBT in patients with anxiety, depression, generalised anxiety disorder and social anxiety disorder (*N* = 174). The study showed high WA scores comparable to face-to-face therapies. They argued that a WA can still be formed in guided digital self-help despite a lack of physical contact over online interactions; agreeing on goals and homework/tasks is essential for successful therapy outcomes^35^. Unlike physical sessions, WA in guided digital therapy is a function of the client’s interaction with the therapist online and access to self-help materials/systems^35^. However, despite the clients improving clinically, there was no association between WA and clinical outcomes; the null association requires further research^35^. Methodological limitations of this study, including a one-off measurement of WA and using an instrument developed for face-to-face therapies, could account for the null association, or maybe WA may not be an active ingredient for guided self-help modalities.

Most of the WA therapeutic-outcome association knowledge is derived from one-to-one therapy delivery. We also set out to understand the effect of WA on group therapy in young people experiencing anxiety and depression. Scoping review evidence produced mixed findings. Group therapy was associated with increased self-esteem, which had a moderating effect on both WA and depression^36^. Group interactions and a secure WA were associated with improved self-esteem and reduced depression^36^. Furthermore, clients with more impaired relational experiences seem to benefit much more from group therapy, signifying a warm WA’s potential impact on treatment outcomes^36^.

Group therapy among adults with mental health problems is also associated with an increase in WA in psychomotor therapies (body awareness and physical activity), with increases in collaboration as the most salient predictor of changes in WA^37^. However, a study by McEvoy et al. (2014) exploring the relationship between interpersonal problems, WA, and outcomes following group (*n* = 115) and individual therapy (*n* = 84), produced slightly different outcomes^26^. In this study, compared to group therapy, individual therapy recipients reported greater WA pre-and post-treatment; the differences were statistically significant^26^. Furthermore, in group therapy, severe pre-treatment anxiety/depression and interpersonal problems were associated with poorer WA and dropout than individual therapy^26^. Some argue that when compared to individual therapy, the group therapy format may not be the most “conducive” platform for clients with severe pre-treatment interpersonal problems to form a functional WA, given their assumed difficulties in relating to other group members and the therapist(s)^2,26^. Clinicians echoed this sentiment during our consultations; nevertheless, more studies are needed to understand the effects of group therapy on WA.

Our review also explored the effect of treatment settings (inpatient vs outpatient) on WA. McLeod and Weisz^27^ carried out a study to explore the relationship between WA and treatment outcomes in youth ((mean age; 10.3 (SD 6.2) years)) with anxiety and depression in an outpatient setting. Their study showed that better child–therapist alliance and parent–therapist alliance during treatment predicted greater reductions in internalising (anxiety and depression) symptoms at the end of treatment. Given that children rarely volunteer to engage in therapy, with parents usually deciding to get involved, a functional WA between therapist(s) and both parents and children is necessary for optimising treatment outcomes^27^. Using a naturalistic study design, Webb et al. (2014) also explored the association between WA and changes in symptomatology in an inpatient setting. For patients with anxiety and depression (*N* = 103) receiving combined CBT and antidepressants in a psychiatry facility^22^, a functional WA was associated with decreased depression. Also, patients with optimism (greater treatment expectations) were likely to form greater WA, subsequently improving treatment outcomes^22^. It seems reasonable to conclude that the WA-outcome association is independent of the setting in which treatment is provided. Further studies are needed to understand the relationship between setting/context and WA-outcome.

The study’s strengths include using a systematic process in searching, retrieving, analysing and synthesising data across sources. More importantly, this study was primarily driven by persons with lived experiences. The involvement of young persons in planning, collecting, analysing, and synthesising the outcomes increases the review’s relevance to the target population of young persons experiencing anxiety and depression.

However, although our review suggests the positive impact of strong WA in treating depression and anxiety, the generalisation of our findings may be limited. Firstly, we did not formally assess the risk of bias in each study. The scoping review aimed to summarise the relationship between WA and mental health outcomes in young people aged 14–24. Future systematic reviews and meta-analyses are warranted. Secondly, most studies were from high-income countries, and their applicability across different settings could be limited; we only retrieved a solitary study from Kenya, a low-income country^34^. There is a need for context-specific studies to explore the effect of WA on anxiety and depression, given the potential influence of culture on WA^3^. We, however, attempted to do so through stakeholder consultations. Thirdly, very few retrieved studies were exclusively done in young adults in the 14–24 age group, which may potentially limit external validity. Finally, albeit the heterogeneous measurement in the WA^2^, there is a need for psychometric evaluation studies to standardise WA measures from diverse perspectives, i.e., patient-, observer- and therapist perspectives^6^.

In conclusion, this review indicates that WA is a salient active ingredient across psychotherapies in managing ongoing anxiety and depression in young persons aged 14–24. Findings from stakeholder consultations and synthesis workshops corroborated this. Although more research is needed to understand WA’s influence in managing anxiety and depression in young people, based on this review and subsequent stakeholder consultations, we recommend routine evaluation of WA from both patients’ and clients’ viewpoints^2,7,37^ at multiple timepoints over therapy. Also, there is a need to explore ways to promote better WA across psychotherapies^2,7^. Lastly, more targeted research using: longitudinal designs, adequately powered experimental designs, multiple WA measurements and advanced statistical modelling techniques to differentiate within- and between-patient differences^1,2,6^ is needed to understand the impact of WA on anxiety and depression in young persons aged 14–24 years.

## Supplementary information

Supplementary Files

## Data Availability

The datasets generated during and/or analysed during the current study are available from the corresponding author on reasonable request.
