# Supplementary material for: The impact of working alliance in managing youth anxiety and depression: a scoping review
Source: Npj Ment Health Res. 2023 Jan 30;2:1. doi: 10.1038/s44184-023-00021-2 (PMC9885927; doi:10.1038/s44184-023-00021-2)
Supplement: Supplementary file 1 — Supplementary Files [file 44184_2023_21_MOESM1_ESM.pdf]

Supplementary Files

Supplementary File 1: Preferred Reporting Items for Systematic reviews and Meta-Analyses extension for Scoping Reviews (PRISMA-ScR) Checklist

SECTION	ITEM	PRISMA-ScR CHECKLIST ITEM	REPORTED ON PAGE #
**TITLE**			
Title	1	Identify the report as a scoping review.	1
**ABSTRACT**			
Structured summary	2	Provide a structured summary that includes (as applicable): background, objectives, eligibility criteria, sources of evidence, charting methods, results, and conclusions that relate to the review questions and objectives.	2
**INTRODUCTION**			
Rationale	3	Describe the rationale for the review in the context of what is already known. Explain why the review questions/objectives lend themselves to a scoping review approach.	3-4
Objectives	4	Provide an explicit statement of the questions and objectives being addressed with reference to their key elements (e.g., population or participants, concepts, and context) or other relevant key elements used to conceptualize the review questions and/or objectives.	4
**METHODS**			
Protocol and registration	5	Indicate whether a review protocol exists; state if and where it can be accessed (e.g., a Web address); and if available, provide registration information, including the registration number.	N/A
Eligibility criteria	6	Specify characteristics of the sources of evidence used as eligibility criteria (e.g., years considered, language, and publication status), and provide a rationale.	4
Information sources*	7	Describe all information sources in the search (e.g., databases with dates of coverage and contact with authors to identify additional sources), as well as the date the most recent search was executed.	4
Search	8	Present the full electronic search strategy for at least 1 database, including any limits used, such that it could be repeated.	5
Selection of sources of evidence†	9	State the process for selecting sources of evidence (i.e., screening and eligibility) included in the scoping review.	5
Data charting process‡	10	Describe the methods of charting data from the included sources of evidence (e.g., calibrated forms or forms that have been tested by the team before their use, and whether data charting was done independently or in duplicate) and any processes for obtaining and confirming data from investigators.	5
Data items	11	List and define all variables for which data were sought and any assumptions and simplifications made.	5
Critical appraisal of individual sources of evidence§	12	If done, provide a rationale for conducting a critical appraisal of included sources of evidence; describe the methods used and how this information was used in any data synthesis (if appropriate).	5
Synthesis of results	13	Describe the methods of handling and summarizing the data that were charted.	5
**RESULTS**			
Selection of sources of evidence	14	Give numbers of sources of evidence screened, assessed for eligibility, and included in the review, with reasons for exclusions at each stage, ideally using a flow diagram.	6-7
Characteristics of sources of evidence	15	For each source of evidence, present characteristics for which data were charted and provide the citations.	7-8
Critical appraisal within sources of evidence	16	If done, present data on critical appraisal of included sources of evidence (see item 12).	N/A
Results of individual sources of evidence	17	For each included source of evidence, present the relevant data that were charted that relate to the review questions and objectives.	7-8
Synthesis of results	18	Summarize and/or present the charting results as they relate to the review questions and objectives.	7-8
**DISCUSSION**			

SECTION	ITEM	PRISMA-ScR CHECKLIST ITEM	REPORTED ON PAGE #
Summary of evidence	19	Summarize the main results (including an overview of concepts, themes, and types of evidence available), link to the review questions and objectives, and consider the relevance to key groups.	10-15
Limitations	20	Discuss the limitations of the scoping review process.	15
Conclusions	21	Provide a general interpretation of the results with respect to the review questions and objectives, as well as potential implications and/or next steps.	15
**FUNDING**			
Funding	22	Describe sources of funding for the included sources of evidence, as well as sources of funding for the scoping review. Describe the role of the funders of the scoping review.	16

JBI = Joanna Briggs Institute; PRISMA-ScR = Preferred Reporting Items for Systematic reviews and Meta-Analyses extension for Scoping Reviews.

* Where *sources of evidence* (see second footnote) are compiled from, such as bibliographic databases, social media platforms, and Web sites.

† A more inclusive/heterogeneous term used to account for the different types of evidence or data sources (e.g., quantitative and/or qualitative research, expert opinion, and policy documents) that may be eligible in a scoping review as opposed to only studies. This is not to be confused with *information sources* (see first footnote).

‡ The frameworks by Arksey and O'Malley (6) and Levac and colleagues (7) and the JBI guidance (4, 5) refer to the process of data extraction in a scoping review as data charting.

§ The process of systematically examining research evidence to assess its validity, results, and relevance before using it to inform a decision. This term is used for items 12 and 19 instead of "risk of bias" (which is more applicable to systematic reviews of interventions) to include and acknowledge the various sources of evidence that may be used in a scoping review (e.g., quantitative and/or qualitative research, expert opinion, and policy document).

From: Tricco AC, Lillie E, Zarin W, O'Brien KK, Colquhoun H, Levac D, et al. PRISMA Extension for Scoping Reviews (PRISMAScR): Checklist and Explanation. Ann Intern Med. 2018;169:467–473. doi: 10.7326/M18-0850.

Supplementary File 2: Research interview guide for clinicians

Section A: Clinician's views on working alliance
Q1. In general, what is your understanding of the term working for alliance (WA)/therapeutic alliance (TA)?
Notes/prompts:

- If the respondent is unsure or provides a definition not necessarily congruent with our working definition, kindly acknowledge their contribution and say the following. **"Thank you for your insights; others have defined WA as " shared confidence that therapy will be helpful and an agreement between the client and therapist over the assignment of therapy tasks. The relationship includes mutual trust and reciprocal liking".**
- If the respondent gives a correct description, proceed to provide the working definition as this is the basis of subsequent questions, i.e., repeat the definition above.

Q2. In what way do you think having/not having a personal connection with a client affects treatment outcomes?
Section B: Questions regarding WA elements (goal, task, bond)]
Q3. What key elements do you think are necessary for the development of a WA?
Note: "Thank you for your insights; from the literature, it is generally agreed that the following three elements are important in developing a WA, i.e., goal, task, and bond. I would wish to understand your views and the importance and how practical it is to attain these elements during counselling sessions. Now, we are going to discuss the three elements one at a time.
Q4. First, let's talk about Goal setting, I will provide the definition first. Goal setting is defines as…*[NOTE provide definition first:* *Goal setting entails the agreement of therapy goals between the counsellor and client.]*
Q4a. Do you think goal setting is important during counselling sessions?
Q4b. Do you think agreeing on goals with a client may influence the development of a WA?
Q4c. How often do you set goals with your clients during counselling sessions?
Second, let's discuss Tasks… Tasks are defined as …. *[NOTE provide definition first:* *Tasks entail the assignment of homework/assignments to the client as part of the therapy process]*
Q5a. Do you think agreement on tasks is important during counselling sessions?
Q5b. Do you think agreeing on a task assignment with a client may influence the development of a WA?
Q5c. How often do you agree on tasks assignment with your clients during counselling sessions?
Last, let's discuss bond… Bond are defined as …. *[NOTE provide definition first:* *Bond entails the development of mutual affection/reciprocal liking between therapists and client during therapy sessions.]*
Q6a. Do you think bond creation is important during counselling sessions?
Q6b. Do you think developing a bond with a client may influence the development of a WA?
Q6c. How often do you develop a bond with your clients during counselling sessions?
Q7. Of these three elements, which one do you consider to be the most important and why?
7b. Ask the therapist to rank the order of perceived important

Importance	Domain, i.e. GOAL, TASK, BOND
1. Most important	
2. Medium importance	
3. Least important	

7c. How does a lack of these elements impact a functional WA?
Section C: Relevance of individual characteristics
8a) Which individual characteristics are necessary for an ideal WA from both patients and therapists? i) Firstly, tell me about the **client's characteristics** necessary for the formation of a WA?
ii) Secondly, what **therapist characteristics** are essential for the development of a WA?
PROMPTS: You may ask about the impact of the following factors if the respondent has not alluded to these factors; 1) age, 2) gender, 3) religion, etc.,?
9. Do you think specific therapist characteristics important in WA development can be learned or inborn, i.e. empathy, congruence, not dominating clients, listening skills etc.?

Thank you for your time. Is there anything else that you would want to comment on? If not, thank you for your time, and we will get in touch with you soon to compensate you for the valuable time committed to this interview.

Supplementary File 3: Research interview guide for patients

<u>Section A: Patient's views on working alliance</u>

Q1. In general, what is your understanding of the term working for alliance (WA)/therapeutic alliance (TA)/personal connection with a therapist?

Notes/prompts:

- If the respondent is unsure or provides a definition not necessarily congruent with our working definition, kindly acknowledge their contribution and say the following. **"Thank you for your insights; others have defined WA as " shared confidence that therapy will be helpful and an agreement between the client and therapist over the assignment of therapy tasks. The relationship includes mutual trust and reciprocal liking".**
- If the respondent gives a correct description, proceed to provide the working definition as this is the basis of subsequent questions, i.e., repeat the definition above.

Q2. In what way do you think having/not having a personal connection with your therapist affects treatment outcomes?

<u>Section B: Questions regarding WA elements (goal, task, bond)</u>

<u>**Note:**</u> Next we are going to talk about elements that have been shown to influence the development of a WA between a client and a therapist.

Q3. What key elements do you think are necessary for the development of a WA/personal connection/collaborative relationship between a client and a counsellor?

Note: "Thank you for your insights; from the literature, it is generally agreed that the following three elements are important in developing a WA, i.e., goal, task, and bond. I would wish to understand your views and the importance and how practical it is to attain these elements during counselling sessions. Now, we are going to discuss the three elements one at a time.

Q4. First, let's talk about <u>BOND</u>. I will provide the definition first. Goal setting is defined as…*[NOTE provide definition first:* *Bond means the development of mutual affection/reciprocal liking between therapists and clients during therapy sessions.*

Q4a. Did you feel that you developed a relationship/bond/personal connection with your counsellor during the therapy sessions?

Q4b. What was (is) the nature of your relation/bond with your counsellor?

Q4c. What do you think about the importance of a relationship or bond between a client and a counsellor?

Q4d. Where there times when you felt that your relationship/bond with was affected? If so, what had happened?

Q4e. What do you think needs to be done to improve the relationship/bond between counsellors and clients?

Q5. Second, let's talk about <u>GOAL SETTING</u>. I will provide the definition first. Goal setting is defines as…*[NOTE provide definition first:* *Goal setting entails the agreement of therapy goals between the counsellor and client.]*

Q5a. To what extend were you involved in deciding on the treatment goals?

Q5b. To what extent do you think being involved in deciding on treatment goals influences treatment outcomes?

Q5c. To what extent do you think being involved in deciding on treatment goals influences the development of a WA?

Q6. Last, let's talk about TASKS. I will provide the definition first. <u>***[NOTE provide definition first:*** *Tasks means the assignment of homework/assignments to the client as part of the therapy process]*</u>

Q6a. To what extent were you involved in deciding on the tasks as part of the counselling process?

Q6b. To what extent do you think being involved in deciding on tasks influences treatment outcomes?

Q6c. To what extent do you think being involved in deciding tasks influences the development of a WA?

7. Of these three elements, which one do you consider to be the most important and why?

Note: Ask the client to rank the order of perceived important

Importance	Domain, i.e. GOAL, TASK, BOND
4. Most important	
5. Medium importance	
6. Least important	

<u>Section C: Relevance of individual characteristics</u>

1a. Which individual characteristics are necessary for the formation of a functional WA for both patients and therapists?

i) Firstly, tell me about the **<u>client's characteristics</u>** necessary for the formation of a WA?

ii) Secondly, what **<u>therapist characteristics</u>** are essential for the development of a WA?

<u>PROMPTS</u>: You may ask about the impact of the following factors if the respondent has not alluded to these factors; 1) age, 2) gender, 3) religion 4) empathy 5) listening therapists etc?

Thank you for your time. Is there anything else that you would want to comment on? If not, thank you for your time, and we will get in touch with you soon to compensate you for the valuable time committed to this interview.
